# Supplementary material for: Analysis of PPARγ Signaling Activity in Psoriasis
Source: Int J Mol Sci. 2021 Aug 10;22(16):8603. doi: 10.3390/ijms22168603 (PMC8395241; doi:10.3390/ijms22168603)
Supplement: Supplementary file 1 [file ijms-22-08603-s001.zip › Supplemental materials_Analysis of PPARg signaling activity in psoriasis/Pathway models/Models images and html files/Interleukin-17 and interleukin-22 signaling in psoriasis/8105.html]

NFKBIA


# Protein NFKBIA

|  |  |
| --- | --- |
| URN | urn:agi-llid:4792 |
| Total Entities | 0 |
| Connectivity | 3982 |
| Name | NFKBIA |
| Description | nuclear factor of kappa light polypeptide gene enhancer in B-cells inhibitor, alpha |
| Notes | This gene encodes a member of the NF-kappa-B inhibitor family, which contain multiple ankrin repeat domains. The encoded protein interacts with REL dimers to inhibit NF-kappa-B/REL complexes which are involved in inflammatory responses. The encoded protein moves between the cytoplasm and the nucleus via a nuclear localization signal and CRM1-mediated nuclear export. Mutations in this gene have been found in ectodermal dysplasia anhidrotic with T-cell immunodeficiency autosomal dominant disease. [provided by RefSeq, Aug 2011] |
| Primary Cell Localization | Cytoplasm |
| Class | Transcription factor |
| ObjectType | Protein |

---

|  |  |
| --- | --- |
| Pathway | Osteoarthritis |
|  | MTHFR Activity Regulation |
|  | Dopamine Receptors Signaling |
|  | Cortisol in Resolving Inflammation |
|  | Toll-like Receptors Act through MYD88-TIRAP Signaling |
|  | Toll-like Receptors Act through MYD88 Signaling |
|  | Dectin-1 (CLEC7A) Signaling |
|  | DC-SIGN (CD209) Signaling |
|  | TLR4 Signaling in Leukocytes |
|  | T-Cell Dependent B-Cell Activation |
|  | T-Cell Independent B-Cell Activation |
|  | V(D)J Recombination Activation |
|  | Immunoglobulin Class-Switch Recombination Activation |
|  | CD8+ T-Cell Activation |
|  | Th1-Cell Differentiation |
|  | Treg-Cell Differentiation |
|  | Th2-Cell Differentiation |
|  | T-Cell Receptor Signaling |
|  | Th17-Cell Differentiation |
|  | AHR Signaling in Tr1 Cells Function |
|  | CXC Chemokine Receptor Signaling |
|  | CC Chemokine Receptor Signaling |
|  | Plasmin Effects in Inflammation |
|  | Proplatelet Maturation |
|  | Bradykinin Effects in Inflammation |
|  | Toll-like Receptors in Sterile Inflammation |
|  | Toll-like Receptor Independent Sterile Inflammation |
|  | MC1R in anti-Inflammatory Signaling |
|  | Prostaglandin E2 Receptor Signaling in Neurons |
|  | Leukotriene Effect on Vascular Endothelial Cell Response |
|  | Eicosanoids in Myeloid Cell Activation |
|  | Basophil Activation |
|  | Eosinophil Survival by Cytokine Signaling |
|  | Eosinophil Activation |
|  | Mast-Cell Activation without Degranulation through TNFSF8 Signaling |
|  | Mast-Cell Activation via IgE Signaling |
|  | Mast-Cell Activation without Degranulation |
|  | Mast-Cell Activation without Degranulation through IL33/IL1RL1 Signaling |
|  | Mast-Cell Activation without Degranulation through KIT/F2R Signaling |
|  | Mast-Cells De Novo Synthesized Mediators via IgE Independent Signaling |
|  | Mast-Cell Activation without Degranulation through IL1R1 and TLR Signaling |
|  | Macrophage M1 Lineage |
|  | CD16/CD14 Proinflammatory Monocyte Activation |
|  | AHR Signaling in M1 Macrophages Function |
|  | anti-Inflammatory Function of Macrophage M2 Lineage |
|  | AXL Receptor Inhibits Macrophages and Dendritic Cells Function |
|  | Neutrophil Recruitment and Priming |
|  | Neutrophil Activation via Adherence on Endothelial Cells |
|  | Negative Acute Phase Proteins Synthesis |
|  | Positive Acute Phase Proteins Synthesis |
|  | Vascular Endothelial Cell Activation by NO |
|  | Vascular Endothelial Cell Activation by Cytokines |
|  | Vascular Endothelial Cell Activation by Blood Coagulation Factors |
|  | Vascular Endothelial Cell Activation by Growth Factors |
|  | Vascular Endothelial Cell Permeability Activation |
|  | Adipokines Production by Adipocyte |
|  | Lipolysis Regulation in Adipocyte |
|  | Nociception Expression Targets Signaling |
|  | BDKRB1/2 -> Interleukins Production |
|  | DRD2 -> TRPC1 Transcription |
|  | TACR1 -> TNF/IL6/IL8 Production |
|  | IL1B Expression Targets -> Nociception |
|  | SCF/BTRC Complex |
|  | SCF/FBXO7 Complex |
|  | Proteins Involved in Atherosclerosis |
|  | Scavenger Receptor OLR1 in Inflammation Related Endothelial Dysfunction |
|  | Dendritic Cells Function in Atherosclerosis |
|  | RAGE/AGER and S100 Proteins in Cardiovascular Injury |
|  | Th1-Cells Activation and Proliferation in Atherosclerosis |
|  | Dilated Cardiomyopathy |
|  | Cardiomyocyte Hypertrophy |
|  | Hypertrophic Cardiomyopathy |
|  | Myocardial Remodeling |
|  | Myocardial Infarction |
|  | MAVS in Antiviral Innate Immune Response in Myocarditis |
|  | Lymphocyte Mediated Myocardial Injury in Myocarditis |
|  | Toll-like Receptors (TLR) in Antiviral Innate Immune Response |
|  | NO Synthesis in Early Vasospasm |
|  | Inflammatory Reaction in Acne Vulgaris |
|  | Keratinocyte Proliferation in Acne Vulgaris |
|  | Atopic Dermatitis Onset |
|  | Mast-Cells Activation in Atopic Dermatitis |
|  | Acute Phase in Atopic Dermatitis |
|  | Atopic Dermatitis |
|  | IL17 Signaling in Psoriasis |
|  | IFN-gamma/TNF-alpha Mediated Cell Proliferation in Psoriasis |
|  | Dendritic Cells Function in Psoriasis |
|  | Apoptotic Keratinocytes Clearance Recession in Systemic Lupus Erythematosus |
|  | Dendritic Cell Activation in Systemic Lupus Erythematosis |
|  | Th0-Cell Aberrant Activation in Systemic Lupus Erythematosus |
|  | Th17-Cell Function in Systemic Lupus Erythematosus |
|  | B-Cell Activation in Systemic Lupus Erythematosus |
|  | Th2-Cell Function in Systemic Lupus Erythematosus |
|  | Acute Cytotoxic CD8+T-Cell Response against Melanocytes |
|  | T-Cell Cytotoxic Response against Melanocytes in Vitiligo |
|  | Humoral Immunity in Vitiligo |
|  | Treg-Cell Function in Diabetes Mellitus Type 1 |
|  | Natural Killer T-Cell Roles in Diabetes Mellitus Type 1 |
|  | non-Suppressive Treg-Cell in Diabetes Mellitus Type 1 |
|  | Plasmacytoid Dendritic Cell in Diabetes Mellitus Type 1 |
|  | TLR2 Signaling in Treg-Cell in Type 1 Diabetes (Animal Model) |
|  | Toll-like Receptors in beta-Cell |
|  | Macrophage Function in Diabetes Mellitus Type 1 |
|  | Peripheral Tolerance to Autoantigens Recession in Diabetes Mellitus Type 1 |
|  | TLR7 and TLR9 in Plasmacytoid Dendritic Cell |
|  | Conventional Dendritic Cell Role in Diabetes Mellitus Type 1 |
|  | Treg-Cell Activation in Diabetes Mellitus |
|  | Advanced Glycation End Products Pathway in Diabetic Microangiopathy |
|  | Protein Kinase C in Diabetic Microangiopathy |
|  | Peripheral Tissue Microangiopathy |
|  | Tubular Cell Dysfunction in Progressive Diabetic Nephropathy |
|  | Podocyte Dysfunction in Diabetic Nephropathy |
|  | Endothelial Cell Dysfunction in Early Diabetic Nephropathy |
|  | Microangiopathy in Diabetic Neuropathy |
|  | Proliferative Diabetic Retinopathy |
|  | AGE Related Insulin Resistance in Skeletal Muscles |
|  | Hepatic Stellate Cells in non-Alcoholic Fatty Liver Disease |
|  | beta-Cell Destruction through Cytokines in Diabetes Mellitus |
|  | beta-Cell Destruction in Diabetes Mellitus Type 1 |
|  | RET1 Mutation in Hyperparathyroidism |
|  | Bone Remodeling in Hyperthyroidism |
|  | Thymic Follicular Hyperplasia |
|  | Fatty Acids in Hypertension and Atherosclerosis Development |
|  | Adipokines Production by Adipocyte Impaired in Obesity |
|  | CD4+T-Cell Response in Celiac Disease |
|  | Enterocyte Mediated Mucosal Damage in Celiac Disease |
|  | CD8+ T-Cell Response in Celiac Disease |
|  | Celiac Disease |
|  | Metastatic Colorectal Cancer |
|  | Crohn's Disease |
|  | Dendritic Cell Dysfunction in Crohn's disease |
|  | OipA Signaling in Helicobacter Pylori Infection |
|  | Proteins Involved in Helicobacter Infections |
|  | CagA Phosphorylation Independent Signaling |
|  | Dendritic Cell Function in Ulcerative Colitis |
|  | Epithelial Cell in the Innate Immune Response in Ulcerative Colitis |
|  | Nicotine anti-Inflammatory Effect in Ulcerative Colitis |
|  | Th2-Cell Function in Ulcerative Colitis |
|  | T-Cell Cytotoxic Response in Ulcerative Colitis |
|  | Antiphospholipid Antibodies in Endothelial Cell |
|  | B-Cell Chronic Lymphocytic Leukemia |
|  | Diffuse Large-B-Cell Lymphoma |
|  | Diffuse Large-B-Cell Lymphoma ABC Subtype |
|  | Primary Mediastinal B-cell Lymphoma (PMBL) Subtype |
|  | Follicular Lymphoma |
|  | Hodgkin Lymphoma |
|  | Mantle Cell Lymphoma |
|  | mTOR/NF-kB/BCR Signaling Disregulation in Mantle Cell Lymphoma |
|  | Mucosa-Associated Lymphoid Tissue (MALT) Lymphoma |
|  | Splenic Marginal Zone Lymphoma |
|  | TNFR/NF-kB Classical Pathway in Multiple Myeloma Plasma Cells |
|  | Osteoclast Activation in Multiple Myeloma |
|  | Multiple Myeloma |
|  | Antiphospholipid Antibodies and Endothelial Cell Interaction |
|  | T-Cell Acute Lymphoblastic Leukemia |
|  | Eosinophil Survival in Asthma |
|  | Il17 Signaling Related Neutrophilia in Asthma |
|  | IL33 Signaling Related Eosinophilia in Asthma |
|  | TSLP Signaling in Bronchial Epithelial Cell in Asthma |
|  | IL17F Signaling in Bronchial Epithelial Cell in Asthma |
|  | IgE Induces Airway Smooth Muscle Cell Proliferation |
|  | Goblet-Cell Related Mucus Secretion in Asthma |
|  | Club-Cell Role in Asthma |
|  | Neutrophil Degranulation via FPR1/IL8 |
|  | Basophil Activation in Asthma |
|  | Mast-Cell Activation in Asthma |
|  | Mast-Cell Related Chemokines Signaling in Asthma |
|  | Antigen-Presenting Cell Role in Asthma |
|  | B-Cell Mediated IgE Production in Asthma |
|  | Th17-Cell Differentiation in Asthma |
|  | Th2-Cell Response in Asthma |
|  | Mucin Production in Goblet Airway Epithelial Cells |
|  | CFTR Facilitates Oxidative Stress in Airway Epithelium in CF |
|  | Airway Epithelial Cell Dysfunction |
|  | CFTR Expression in Epithelial Cells (Class I Mutations) |
|  | Osteoclast Activation in Gout |
|  | Neutrophil Recruitment in Sinovium in Gout |
|  | Monocytes Function in Gout |
|  | Hyper IgM Syndrome |
|  | TNF and IL1B Induce Metalloproteinase Synthesis in Osteoarthritis |
|  | Apoptosis of Chondrocyte |
|  | IL1B Induced Arthralgia |
|  | Dendritic Cell Dysfunction in Psoriatic Arthritis |
|  | Th17-Cell and Th1 Immune Responsein Psoriatic Arthritis |
|  | Osteoclast Activation in Psoriatic Arthritis |
|  | Synovial Fibroblast Activation in Psoriatic Arthritis |
|  | Keratinocyte Activation in Psoriatic Arthritis |
|  | Proteins Involved in Psoriatic Arthritis |
|  | PTPN22 Role in Rheumatoid Arthritis |
|  | Osteoclast Activation in Rheumatoid Arthritis |
|  | Synovial Fibroblast Activation by Citokines in Rheumatoid Arthritis |
|  | Apoptosis Block in Synovial Fibroblast |
|  | TLR2 Induced Synovial Fibroblast Activation in Rheumatoid Arthritis |
|  | Autocrine Cytokine/Chemokine Loops in Systemic Scleroderma |
|  | B-Cells Function in Systemic Scleroderma |
|  | Th2-Cells Function in Systemic Scleroderma |
|  | Skin Fibrosis |
|  | Proteins Involved in HIV Type 1 Infection |
|  | CD4+ T-Cell Function Decline in HIV |
|  | Apoptosis Block in Infected Cells |
|  | CD4+ T-Cell Death in HIV |
|  | Toll-like Receptors (TLR) in Periodontitis |
|  | Diabetes Induced Periodontitis |
|  | IL1 Receptor Antagonist (IL1RN) Deficiency (DIRA) |
|  | PAPA Syndrome |
|  | TNF Receptor Associated Periodic Syndrome |
|  | Mevalonate Kinase Deficiency |
|  | Blau Syndrome |
|  | IL36 Receptor Antagonist (IL1F5) Deficiency (DITRA) |
|  | Cryopyrin (NLPR3) Associated Periodic Syndromes |
|  | Familial Cold Autoinflammatory Syndrome 2 |
|  | Familial Mediterranean Fever |
|  | Majeed Syndrome |
|  | Glutamate Toxiicity Effects on Striatum Medium Spiny Neurons in Hantington's Disease |
|  | NF-kB Signaling Activation in Huntington Disease |
|  | Gluten Impact on Neuronal System (Hypothesis) |
|  | IL6 Promotes Inflammation in Muscular Dystrophies |
|  | Muscular Dystrophies |
|  | Dystrophin Glycoprotein Complex Signaling in Muscular Dystrophies |
|  | Osteoclasts Function in Osteopetrosis |
|  | Osteoclast Activation in Postmenopause |
|  | Genes with Mutations Associated with Psoriatic Arthritis |
|  | Microglia Activation in Alzheimer's Disease |
|  | HMGB1 and IL1B in Neuroinflammation |
|  | Proteins Involved in Glioblastoma |
|  | Vascular Endothelial Cell Vasodilation in Brain |
|  | Postsynaptic Neuron Activation |
|  | nNOS (NOS1) and iNOS (NOS2) Activation via Glutamate |
|  | Growth Factor Signaling in Neuroblastoma |
|  | Neuroblastoma |
|  | Microglia Activation in Parkinson's Disease |
|  | Glutamate Role in Parkinson's Disease |
|  | Glutamate Mediated Excitotoxicity in Amyotrophic Lateral Sclerosis |
|  | Crystallin alpha B (CRYAB) Suppresses Multiple Sclerosis |
|  | MIR155 Role in Th17-Cell in Multiple Sclerosis |
|  | SPP1 Induced Survival of Myelin Specific T-Cells in Multiple Sclerosis |
|  | Proteins Involved in Breast Cancer Related to NOTCH Signaling Pathway |
|  | Breast Cancer |
|  | Coagulation Factors and PAR1/2 Receptors (F2R/F2RL1) in Endometriosis |
|  | MIF Role in Cholesteatoma |
|  | EGF/cyclinD1 Signaling in Acquired Middle Ear Cholesteatoma |
|  | HMGB1/RAGE Signaling in Middle Ear Cholesteatoma |
|  | Subepithelial Fibroblasts in Middle Ear Cholesteatoma |
|  | Cholesteatoma |
|  | HPV E6 and E7 Induced Disruption of TNFR/NF-kB Signaling in Keratinocytes |
|  | TLR3 Role in Retinal Pigment Epithelial Cell |
|  | EPHA2 Mutations in Cataract |
|  | OPTN Effects on NF-kB Signaling in Glaucoma |
|  | Glutamate Mediated Excitotoxicity in Glaucoma |
|  | Neurotrophic Factor Deprivation in Retinal Ganglion Cell Death |
|  | Cytokines Trigger Otitis Media |
|  | Immune System Role in Otitis Media |
|  | Alveolar Macrophage Negative Regulation Declined in COPD |
|  | Alveolar Macrophages Dysfunction in COPD |
|  | T-Cell Cytotoxic Activation |
|  | Alveolar Epithelial Cell Dysfunction in COPD |
|  | Mucin Hyperproduction in Goblet and Mucous Cells |
|  | Advanced Glycation End Products in Diabetes or Age Related Erectile Dysfunction |
|  | Plasmin Role in Glomerulonephritis |
|  | Endothelial Cell Dysfunction in Glomerulonephritis |
|  | Toll-like Receptors in Pyelonephritis |
|  | Endothelial Cell Dysfunction in Pyelonephritis |
|  | Ammonia Effects on Brain Cells |
|  | CD157/ITGB2 Signaling in Myeloid Cell |
|  | CD38/CD3 -> JUN/FOS/NF-kB Signaling in T-cell Proliferation |
|  | CD38/CD19 -> JUN/FOS/NF-kB Signaling in B-cell Proliferation |
|  | S100P Expression Target |
|  | HMGB1 Expression Targets |
|  | TTR Expression Targets |
|  | S100B Expression Targets |
|  | Dopamine/Gi Expression Targets |
|  | B-Cell Receptor -> NF-kB Signaling |
|  | TGFBR3 -> ARRB2 Signaling |
|  | CD19 Expression Targets |
|  | CD81 -> AP-1 Expression Targets |
|  | gamma Globulins Expression Targets |
|  | CD22 -> NF-kB Expression Targets |
|  | CD19/CR2 Expression Targets |
|  | CD72 -> STAT Expression Target |
|  | CD8 -> STAT Expression Targets |
|  | CD80 -> STAT Expression Targets |
|  | CD86 -> STAT Expression Targets |
|  | CSF2 -> NF-kB Expression Targets |
|  | Doxorubicin Induced Cardiotoxicity |
|  | Clozapine Induced Granulocytopenia |
|  | Cyclosporine Induced Nephrotoxicity |
|  | EGF -> CTNN Expression Targets |
|  | Erythropoietin -> NF-kB Expression Targets |
|  | PECAM1 Expression Targets |
|  | F2 -> STAT1/NF-kB Expression Targets |
|  | PLG -> STAT1/NF-kB Expression Targets |
|  | EGFR -> Expression Targets in Colon |
|  | GH1/GHR -> NF-kB/ELK/SRF/MYC Expression Targets |
|  | CSH1/GHR Expression Targets |
|  | NFKB Canonical Signaling Activation in Cancer |
|  | STAT3 and NFkB Activate Inflammation-Induced Tumorigenesis |
|  | IL23A and IL17A Provoke Cancer-Associated Inflammation |
|  | Genes with Mutations in Cancer-Associated Evading of Growth Suppressors |
|  | Proteins with Altered Expression in Cancer-Associated Evading of Growth Suppressors |
|  | NFKB Signaling Activation by Blocking of Tumor Suppressors |
|  | Proteins with Altered Expression in Cancer-Associated Resisting to Cell Death |
|  | Genes with Mutations in Cancer-Associated Resisting to Cell Death |
|  | Apoptosis Block by Death Receptor Signaling in Cancer |
|  | Apoptosis Evasion in Cancer: Overview |
|  | Telomeric Proteins/NFKB Positive Feedback Interplay in Cancer |
|  | Oxidized LDL/OLR1 Activates Cell Proliferation in Cancer |
|  | ANGPT/TEK Maintains Endothelial Cell Quiescence in Cancer |
|  | Cancer Cells Inhibit Adipocyte Differentiation |
|  | Skeletal Muscle Wasting in Cancer Cachexia |
|  | Lactate as a Signaling Molecule in Cancer Cells |
|  | Treg Cells Promote Immunosuppression in Cancer Immune Escape |
|  | IDO1 in Cancer Immune Escape |
|  | Effector T-cell Inactivation in Cancer Immune Escape |
|  | CD19 -> NF-kB Signaling |
|  | AGER -> NF-kB Signaling |
|  | FibronectinR -> NF-kB Signaling |
|  | MacrophageR -> CEBPB -> NF-kB Signaling |
|  | Fibronectin Expression Targets |
|  | ICAM1 Expression Targets |
|  | Fibrinogen Expression Targets |
|  | EGFR -> Expression Targets in Skin |
|  | IFNLR1-> STAT/NFKB Signaling |
|  | IL7R -> FOXO/NF-kB Signaling |
|  | IL15R -> NF-kB/NFATC Signaling |
|  | IL23R -> STAT3/NF-kB Signaling |
|  | IL1R -> NF-kB Signaling |
|  | IL12R -> NF-kB/NFATC Signaling |
|  | IL7 Expression Targets |
|  | IL15 Expression Targets |
|  | IL12B Expression Targets |
|  | IL16 -> NF-kB Expression Targets |
|  | PAF/Gq -> NF-kB Expression Targets |
|  | CD40 -> Expression Targets in Lymph Node |
|  | Elevated Receptors -> Expression Targets in Spleen |
|  | NCAM1 -> Expression Targets in Nerve Tissue |
|  | NGF -> SMAD3/NF-kB Expression Targets |
|  | BDNF Expression Targets |
|  | NTF3 Expression Targets |
|  | NTF4 Expression Targets |
|  | NOTCH -> NF-kB Signaling |
|  | DLL4 Expression Targets |
|  | ADAM17 Expression Targets |
|  | JAG1 Expression Targets |
|  | DLL1 Expression Targets |
|  | NOTCH Expression Targets |
|  | Adenosine Expression Targets |
|  | PRL/GHR -> NF/kB/ELK/SRF/MYC Expression Targets |
|  | PDCD1 -> NF-kB Expression Targets |
|  | DDR1 -> NF-kB Signaling |
|  | Collagen -> NF-kB Expression Targets |
|  | TNF-alpha/TNFRSF1A Signaling |
|  | TNF-alpha/TNFRSF1B Signaling |
|  | NF-kB Canonical Signaling |
|  | TCR -> NF-kB Expression Targets |
|  | T-Cell Receptor -> NF-kB Signaling |
|  | FASLG Expression Targets |
|  | TNF -> NF-kB Expression Targets |
|  | TNFSF10 Expression Targets |
|  | LTA Expression Targets |
|  | TNFSF14 Expression Targets |
|  | CD40LG -> NF-kB/ELK/SRF -> CREB/NFATC Expression Targets |
|  | TNFSF13 Expression Targets |
|  | TNFSF13B Expression Targets |
|  | EDA Expression Targets |
|  | EctodysplasinR -> NF-kB Signaling |
|  | NGFR -> NF-kB Signaling |
|  | TNFR -> NF-kB Signaling |
|  | TLR3 Expression Targets |
|  | TLR5 Expression Targets |
|  | TLR7 Expression Targets |
|  | TLR1 -> 2/6 Expression Targets |
|  | IL1B Expression Targets |
|  | TLR4 -> NF-kB/IRF Expression Targets |
|  | IL1A Expression Targets |
|  | TLR9 Expression Targets |
|  | TLR4/5/7/9 -> NF-kB Signaling |
|  | TLR1/2/6 -> NF-kB Signaling |
|  | TLR3 -> NF-kB Signaling |
|  | ROS in Triggering Vascular Inflammation |
|  | Glutamate Mediated Neurotoxicity |
|  | Ca2+ Cytosolic Overload |
|  | pathway 4 |
|  | cypa |
|  | prarg negative regulators, ps-positive |
|  | PPARG negative regulators |
|  | pparg expr target\_regulators |
|  | Neighbors of overall survival |
|  | Neighbors of disease severity |
|  | Neighbors of tumor size |
|  | Neighbors of lesion size |
|  | Neighbors of infarct size |
|  | Neighbors of insulin sensitivity |
|  | Neighbors of bacterial load |
|  | Neighbors of cancer survival |
|  | Neighbors of graft survival |
|  | Neighbors of lethality |
|  | Neighbors of tumor regression |
|  | Neighbors of body weight |
|  | Neighbors of microvessel density |
|  | Neighbors of radioresistance |
|  | Neighbors of shear stress |
|  | Neighbors of viral load |
|  | Neighbors of cancer risk |
|  | Neighbors of cancer incidence |
|  | Neighbors of blood glucose |
|  | Neighbors of radiosensitivity |
|  | Neighbors of cell permeability |
|  | Neighbors of gastric cancer risk |
|  | Neighbors of infarct area |
|  | Neighbors of lymphocyte count |
|  | Neighbors of overall survival |
|  | Neighbors of disease severity |
|  | Neighbors of tumor size |
|  | Neighbors of lesion size |
|  | Neighbors of infarct size |
|  | Neighbors of insulin sensitivity |
|  | Neighbors of bacterial load |
|  | Neighbors of cancer survival |
|  | Neighbors of graft survival |
|  | Neighbors of lethality |
|  | Neighbors of tumor regression |
|  | Neighbors of body weight |
|  | Neighbors of microvessel density |
|  | Neighbors of radioresistance |
|  | Neighbors of shear stress |
|  | Neighbors of viral load |
|  | Neighbors of cancer risk |
|  | Neighbors of cancer incidence |
|  | Neighbors of blood glucose |
|  | Neighbors of radiosensitivity |
|  | Neighbors of cell permeability |
|  | Neighbors of gastric cancer risk |
|  | Neighbors of infarct area |
|  | Neighbors of lymphocyte count |
|  | Neighbors of cell development |
|  | Neighbors of immune response |
|  | Neighbors of cell population |
|  | Neighbors of T-cell development |
|  | Neighbors of inflammatory response |
|  | Neighbors of angiogenesis |
|  | Neighbors of innate immune response |
|  | Neighbors of macrophage activation |
|  | Neighbors of cell formation |
|  | Neighbors of osteoclast development |
|  | Neighbors of epithelial to mesenchymal transition |
|  | Neighbors of chemotaxis |
|  | Neighbors of dendritic cell differentiation |
|  | Neighbors of cell survival |
|  | Neighbors of osteoclast differentiation |
|  | Neighbors of cell phenotype |
|  | Neighbors of T-cell proliferation |
|  | Neighbors of cell adhesion |
|  | Neighbors of T-cell activation |
|  | Neighbors of tumor growth |
|  | Neighbors of dendritic cell development |
|  | Neighbors of cell count |
|  | Neighbors of cell infiltration |
|  | Neighbors of SMC proliferation |
|  | Neighbors of endothelial cell migration |
|  | Neighbors of adipogenesis |
|  | Neighbors of lipid storage |
|  | Neighbors of cell damage |
|  | Neighbors of aging |
|  | Neighbors of cell motility |
|  | Neighbors of bone resorption |
|  | Neighbors of cell migration |
|  | Neighbors of smooth muscle cell migration |
|  | Neighbors of hepatic regeneration |
|  | Neighbors of transendothelial migration |
|  | Neighbors of phagocytosis |
|  | Neighbors of leukocyte migration |
|  | Neighbors of leukocyte recruitment |
|  | Neighbors of cell invasion |
|  | Neighbors of heart function |
|  | Neighbors of immune system function |
|  | Neighbors of autophagy |
|  | Neighbors of monocyte adhesion |
|  | Neighbors of transcription activation |
|  | Neighbors of senescence |
|  | Neighbors of hepatocyte proliferation |
|  | Neighbors of neuroprotection |
|  | Neighbors of neuronal death |
|  | Neighbors of cell growth |
|  | Neighbors of ROS generation |
|  | Neighbors of first trimester pregnancy |
|  | Neighbors of sensitization |
|  | Neighbors of cellular senescence |
|  | Neighbors of colony formation |
|  | Neighbors of cell differentiation |
|  | Neighbors of life span |
|  | Neighbors of inflammatory disease |
|  | Neighbors of rheumatoid arthritis |
|  | Neighbors of atherosclerosis |
|  | Neighbors of fibrosis |
|  | Neighbors of colitis |
|  | Neighbors of injury |
|  | Neighbors of atherogenesis |
|  | Neighbors of carcinogenesis |
|  | Neighbors of inflammation |
|  | Neighbors of obesity |
|  | Neighbors of vascular disease |
|  | Neighbors of diabetes mellitus |
|  | Neighbors of asthma |
|  | Neighbors of pneumonia |
|  | Neighbors of hyperplasia |
|  | Neighbors of osteoarthritis |
|  | Neighbors of leukocyte infiltration |
|  | Neighbors of reperfusion injury |
|  | Neighbors of insulin resistance |
|  | Neighbors of infection |
|  | Neighbors of preeclampsia |
|  | Neighbors of liver injury |
|  | Neighbors of neuroinflammation |
|  | Neighbors of sepsis |
|  | Neighbors of death |
|  | Neighbors of encephalomyelitis |
|  | Neighbors of dermatitis |
|  | Neighbors of neutrophil infiltration |
|  | Neighbors of heart disease |
|  | Neighbors of lung injury |
|  | Neighbors of immunopathology |
|  | Neighbors of hepatocellular carcinoma |
|  | Neighbors of acute lung injury |
|  | Neighbors of gastric cancer |
|  | Neighbors of atherosclerotic plaque |
|  | Neighbors of colorectal cancer |
|  | Neighbors of ischemia |
|  | Neighbors of melanoma |
|  | Neighbors of cancer |
|  | Neighbors of cerebral ischemia |
|  | Neighbors of neoplasm |
|  | Neighbors of neointima |
|  | Neighbors of vasculitis |
|  | Neighbors of atrophy |
|  | Neighbors of myocardial fibrosis |
|  | Neighbors of prostate cancer |
|  | Neighbors of osteolysis |
|  | Neighbors of ovarian cancer |
|  | Neighbors of hypertension |
|  | Neighbors of intimal hyperplasia |
|  | Neighbors of proteinuria |
|  | Neighbors of epithelial cell |
|  | Neighbors of macrophage |
|  | Neighbors of intestine epithelium cell |
|  | Neighbors of neutrophil |
|  | Neighbors of lung |
|  | Neighbors of kidney |
|  | Neighbors of skin |
|  | Neighbors of intestine |
|  | Neighbors of heart |
|  | Neighbors of NF-kB family |
|  | Neighbors of oxidized LDL |
|  | Neighbors of JNK |
|  | Neighbors of IL1 family |
|  | Neighbors of PPAR |
|  | Neighbors of Jun/Fos |
|  | Neighbors of NFKBI |
|  | Neighbors of PKC |
|  | Neighbors of PI3K |
|  | Neighbors of mitogen-activated protein kinase |
|  | Neighbors of IFNAR ligand |
|  | Neighbors of transforming growth factor |
|  | Neighbors of TLR |
|  | Neighbors of cytokine |
|  | Neighbors of interferon |
|  | Neighbors of Ras GTPase |
|  | Neighbors of growth factor |
|  | Neighbors of PKA |
|  | Neighbors of ovalbumin |
|  | Neighbors of beta adrenoceptor |
|  | Neighbors of IKBK |
|  | Neighbors of protein tyrosine kinase |
|  | Neighbors of NAD(P)H oxidase |
|  | Neighbors of IgG |
|  | Neighbors of SMAD subfamily |
|  | Neighbors of AMPK |
|  | Neighbors of p38 |
|  | Neighbors of inflammatory cytokine |
|  | Neighbors of estrogen receptor |
|  | Neighbors of T-cell receptor |
|  | Neighbors of PDGF-BB |
|  | Neighbors of IL12 |
|  | Neighbors of Notch |
|  | Neighbors of heat shock protein 90 |
|  | Neighbors of TSH |
|  | Neighbors of endothelin |
|  | Neighbors of proteasome endopeptidase complex |
|  | Neighbors of class nuclear receptor with C4 zinc fingers |
|  | Neighbors of histone H3 |
|  | Neighbors of SAPK |
|  | Neighbors of PP2B |
|  | Neighbors of CD3 complex |
|  | Neighbors of relaxin |
|  | Neighbors of PDGF receptor |
|  | Neighbors of caspase |
|  | Neighbors of TNF-alpha receptor |
|  | sars-cov no binding |
|  | BCL6-TP53-BCL2 |
|  | dsRNA induces innate immunity and INFs in alveolar cells |
|  | Innate immunity in Leukocytes |
|  | Dendritic Cell Activation in COVID19 |
|  | Figure 3\_Mixed network of dexamethasone targets |
|  | Figure 3\_Mixed network of dexamethasone targets |
|  | 1\_Inflammation in keratinocytes in the pathology of acne |
|  | 2\_Acute phase initiation in atopic dermatitis |
|  | 2\_Interleukin-17 and interleukin-22 signaling in psoriasis |
|  | 1\_The onset of atopic dermatitis |
|  | 1\_TNF and IL1B provoke ECM degradation in osteoarthritis |
|  | 1\_Osteoclast-mediated bone resorption in postmenopausal women |
|  | 1\_Osteoclast functional impairment in osteopetrosis |
|  | 2\_Chondrocyte death in osteoarthritis |
|  | 1\_The role of Toll-like receptors in pyelonephritis |
|  | 2\_The role of endothelial cells in pyelonephritis |
|  | 3\_Role of neurotrophic factor deprivation in retinal ganglion cell death |
|  | 1\_Insufficient activation of immune response in the middle ear epithelium cells in otitis media |
|  | 2\_Pathogens stimulate mucins expression in the middle ear |
|  | 2\_Oxidized LDL causes endothelial dysfunction in atherosclerosis |
|  | 2\_Cardiomyocyte hypertrophy |
|  | 1\_Cigarette smoke causes dysfunction and production of inflammatory mediators in alveolar epithelial cells |
|  | 3\_Role of alveolar macrophages in pulmonary tissue destruction in COPD |
|  | 1\_Th2 cell response in asthma |
|  | 3\_2\_Regulation of airway surface liquid (ASL) thickness |
|  | 3\_1\_Mucus accumulation in asthma |
|  | 2\_2\_Eosinophilia and neutrophilia in asthma: eosinophil apoptosis |
|  | 4\_2\_Airway smooth muscle cells proliferation and airway tissue remodeling |
|  | 4\_Mucin hyperproduction in goblet and mucous cells in COPD |
|  | 2\_Paneth cell dysfunction in Crohn's disease |
|  | 1\_Defects in response to pathogens in the gut promotes inflammation in Crohn's disease |
|  | 3\_Autophagy dysfunction in Crohn's disease |
|  | 2\_Intestine epithelial cells drive immunological responses in ulcerative colitis |
|  | 1\_Intestine epithelial cell dysfunction in ulcerative colitis |
|  | 4\_1\_Polymorphisms associated with inflammatory bowel diseases |
|  | 2\_Lipotoxicity in NAFLD |
|  | 1\_CD4+ T cell and epithelial cell response in celiac disease |
|  | 6\_p12I Viral protein promotes T-cell activation in HTLV-1 infection |
|  | 5\_Human papilloma virus E6 and E7 proteins promote epithelial cell proliferation |
|  | 2\_HIV - induced depletion of CD4+ T-cells |
|  | 1\_CFTR expression and degradation in epithelial cells in cystic fibrosis (class I-II mutations) |
|  | 1\_Antigen-presenting cells promote islet dysfunction and immune system activation in T1D |
|  | 3\_Islet beta-cell destruction in T1D |
|  | 1\_Microangiopathy in diabetic neuropathy |
|  | 2\_Neuron and Schwann cell death |
|  | 1\_Glutamate and calcium excitotoxicity of motor neurons in ALS |
|  | 1\_1\_Glutamate overload enforced prostaglandins and NO synthesis in neurons |
|  | Figure 4\_Diabetes mellitus Type 1: conventional dendritic cell |
|  | drug-target pairs direct regulation |
|  | mapk1 protein modificators |
|  | Interleukin-17 and interleukin-22 signaling in psoriasis |

---

|  |  |
| --- | --- |
| Group | Genes with Mutations Associated with Ageing (Mammalian Animal Model) |
|  | Genes with Mutations Associated with Hodgkin Lymphoma |
|  | Genes with Mutations Associated with Psoriatic Arthritis |
|  | Genes with Mutations Associated with Glioma |
|  | other partners for SARS |

---

|  |  |
| --- | --- |
| Source | Biopax |
|  | curated |
|  | ResNet |
|  | GO component |

---

|  |  |
| --- | --- |
| MedScan ID | 4792 |

---

|  |  |
| --- | --- |
| LocusLink ID | 4792 |
|  | 18035 |
|  | 25493 |
|  | 104829 |
|  | 282291 |
|  | 323099 |

---

|  |  |
| --- | --- |
| Alias | RL/IF-1 |
|  | IkBalpha |
|  | Nuclear factor of kappa light chain gene enhancer in B-cells |
|  | major histocompatibility complex enhance-binding protein MAD3 |
|  | MAD-3 |
|  | Inhibitor of nuclear factor of kappa light chain gene enhancer in B-cells alpha |
|  | P-I-kappaB-alpha |
|  | nuclear factor of kappa light chain gene enhancer in B-cells inhibitor, alpha |
|  | IKBA\_HUMAN |
|  | IkappaBalpha |
|  | NF-kappaB inhibitor alpha |
|  | NFKBIA |
|  | IKBA |
|  | nuclear factor of kappa light polypeptide gene enhancer in B-cells inhibito |
|  | IkappaB/MAD3 |
|  | Inhibitor of 3000097 |
|  | AI462015 |
|  | nuclear factor kappaB inhibitor alpha |
|  | MAD-III |
|  | nuclear factor of kappa light polyp gene enhancer in B-cell I |
|  | NFKB inhibitor alpha |
|  | Major histocompatibility complex enhancer- binding protein MAD3 |
|  | I(Kappa)B(alpha) |
|  | RL/IF I |
|  | MAD-3/I kappa B alpha |
|  | NFKBI |
|  | nuclear factor of kappa light chain gene enhancer in b-cells inhibitor |
|  | IkappaB-alpha/MAD3 |
|  | nuclear factor of kappa light polyp gene enhancer in B-cell 1 |
|  | EDAID2 |
|  | I-kappa-B-alpha |
|  | ikB-alpha |
|  | major histocompatibility complex enhancer-binding protein MAD3 |
|  | nuclear factor of kappa light polypeptide gene enhancer in B-cells inhibitor, alpha |
|  | NF-kappa-B inhibitor alpha |
|  | I-kappaBalpha |
|  | Inhibitor of nuclear factor of kappa light chain gene enhancer in B-cells, alpha |
|  | Ikappa-Balpha |
|  | RL/IF-1s |
|  | lkappa-Balpha |
|  | expressed sequence AI462015 |
|  | RL/IF Is |
|  | I-kappaB-alpha |
|  | MAD III |
|  | MGC56666 |
|  | NF-kappaB inhibitor alpha-like protein B |
|  | alpha b |
|  | chromosome |
|  | fb81f08 |
|  | ikbaB |
|  | nfkbiab |
|  | nuclear factor of kappa light polypeptide gene enhancer in B-cells inhibitor, alpha b |
|  | scaffold |
|  | wu:fb81f08 |
|  | zgc:56666 |
|  | zgc:77616 |

---

|  |  |
| --- | --- |
| GO ID | 0051059 |
|  | 0019899 |
|  | 0008139 |
|  | 0008134 |
|  | 0031625 |
|  | 0007249 |
|  | 0006915 |
|  | 0070417 |
|  | 0007253 |
|  | 0042994 |
|  | 0070498 |
|  | 0031663 |
|  | 0043392 |
|  | 0032088 |
|  | 0045746 |
|  | 0043066 |
|  | 0010888 |
|  | 0010745 |
|  | 0045638 |
|  | 0070427 |
|  | 0070431 |
|  | 0032270 |
|  | 0010875 |
|  | 0050729 |
|  | 0045944 |
|  | 0016579 |
|  | 0006606 |
|  | 1901222 |
|  | 0042127 |
|  | 0043330 |
|  | 0032495 |
|  | 0035994 |
|  | 0034142 |
|  | 0033209 |
|  | 0016032 |
|  | 0033256 |
|  | 0005737 |
|  | 0005829 |
|  | 0005654 |
|  | 0005634 |
|  | 0005886 |
|  | 0031072 |
|  | 0044877 |
|  | 0071345 |
|  | 0071407 |
|  | 0071356 |
|  | 0045893 |
|  | 0010468 |
|  | 0032496 |
|  | 0032991 |
|  | 0097421 |
|  | 0005622 |
|  | 0032403 |
|  | 0045087 |
|  | 0034146 |
|  | 0006916 |
|  | 0071216 |
|  | 0032481 |
|  | 0002756 |
|  | 0002755 |
|  | 0000060 |
|  | 0005515 |
|  | 0034134 |
|  | 0034138 |
|  | 0035666 |
|  | 0008063 |
|  | 0034130 |
|  | 0051092 |
|  | 0038095 |
|  | 0043234 |
|  | 0042345 |
|  | 0048011 |
|  | 0034166 |
|  | 0050852 |
|  | 0038123 |
|  | 0038124 |
|  | 0034162 |
|  | 0019048 |
|  | 0042802 |
|  | 0002224 |
|  | 0002223 |
|  | 0044419 |
|  | 0010552 |
|  | 0045833 |
|  | 0032376 |
|  | 0009618 |
|  | 0009617 |
|  | 0045941 |
|  | 0003719 |

---

|  |  |
| --- | --- |
| KEGG ID | hsa:4792 |
|  | mmu:18035 |
|  | rno:25493 |

---

|  |  |
| --- | --- |
| MedLine Reference | 12200125 |
|  | 12700242 |
|  | 12897149 |
|  | 14764603 |
|  | 15308505 |
|  | 16081881 |
|  | 16135962 |
|  | 16938483 |
|  | 1741294 |
|  | 10195896 |
|  | 10199915 |
|  | 10349636 |
|  | 10702415 |
|  | 10922068 |
|  | 11042159 |
|  | 11067893 |
|  | 11076861 |
|  | 11130978 |
|  | 11133232 |
|  | 11217851 |
|  | 11221845 |
|  | 11241200 |
|  | 11799106 |
|  | 11880364 |
|  | 11913973 |
|  | 12083419 |
|  | 12167702 |
|  | 12397017 |
|  | 12424381 |
|  | 12466851 |
|  | 12477932 |
|  | 12589049 |
|  | 12843402 |
|  | 12896983 |
|  | 12920239 |
|  | 12938156 |
|  | 1339305 |
|  | 14585967 |
|  | 14665694 |
|  | 14741691 |
|  | 14763901 |
|  | 14764534 |
|  | 14767066 |
|  | 14960276 |
|  | 14966265 |
|  | 14978074 |
|  | 14991073 |
|  | 15020231 |
|  | 15102471 |
|  | 15251865 |
|  | 15294950 |
|  | 15317747 |
|  | 15536134 |
|  | 15618518 |
|  | 15671248 |
|  | 15714207 |
|  | 15811852 |
|  | 15820254 |
|  | 15845452 |
|  | 15879100 |
|  | 15923614 |
|  | 15998793 |
|  | 16141072 |
|  | 16141073 |
|  | 16166517 |
|  | 16199483 |
|  | 16231352 |
|  | 16260626 |
|  | 16371367 |
|  | 16452166 |
|  | 16481354 |
|  | 16737960 |
|  | 16804897 |
|  | 16931600 |
|  | 1740450 |
|  | 7552992 |
|  | 7590249 |
|  | 7603567 |
|  | 7867065 |
|  | 7936653 |
|  | 7963540 |
|  | 8628301 |
|  | 8642348 |
|  | 8662753 |
|  | 8858144 |
|  | 8864120 |
|  | 8887559 |
|  | 8914937 |
|  | 9186501 |
|  | 9597130 |
|  | 9743524 |
|  | 9854797 |
|  | 10026139 |
|  | 10085062 |
|  | 10102628 |
|  | 10329681 |
|  | 10393859 |
|  | 10398585 |
|  | 10421793 |
|  | 10480634 |
|  | 10521480 |
|  | 10580107 |
|  | 10582246 |
|  | 10635328 |
|  | 10637284 |
|  | 10644755 |
|  | 10655476 |
|  | 10657303 |
|  | 10723127 |
|  | 10747850 |
|  | 10783893 |
|  | 10848576 |
|  | 10869418 |
|  | 10928981 |
|  | 10969074 |
|  | 11042193 |
|  | 11095741 |
|  | 11106428 |
|  | 11278695 |
|  | 11287411 |
|  | 11313474 |
|  | 11493922 |
|  | 11511100 |
|  | 11696595 |
|  | 11809792 |
|  | 11983170 |
|  | 12028809 |
|  | 12084717 |
|  | 12086926 |
|  | 12193729 |
|  | 12244195 |
|  | 12297126 |
|  | 12377412 |
|  | 12388747 |
|  | 12419806 |
|  | 12429743 |
|  | 12433922 |
|  | 12518988 |
|  | 12620896 |
|  | 12651903 |
|  | 12704657 |
|  | 12711606 |
|  | 12748192 |
|  | 12921778 |
|  | 12944982 |
|  | 12972430 |
|  | 12973300 |
|  | 13680285 |
|  | 1427874 |
|  | 14523047 |
|  | 14561767 |
|  | 14576361 |
|  | 14623898 |
|  | 14685242 |
|  | 14743216 |
|  | 1493333 |
|  | 15068390 |
|  | 15073126 |
|  | 15125834 |
|  | 15161102 |
|  | 15173174 |
|  | 15184376 |
|  | 15202778 |
|  | 15247916 |
|  | 15330761 |
|  | 15337789 |
|  | 15389589 |
|  | 15389633 |
|  | 15613239 |
|  | 15671028 |
|  | 15979856 |
|  | 16046415 |
|  | 16105840 |
|  | 16189514 |
|  | 16258173 |
|  | 16407234 |
|  | 16426569 |
|  | 16756995 |
|  | 16919536 |
|  | 16955245 |
|  | 17003112 |
|  | 17062574 |
|  | 17148610 |
|  | 1829648 |
|  | 3140380 |
|  | 7479976 |
|  | 7565415 |
|  | 7679069 |
|  | 7700645 |
|  | 7739562 |
|  | 7878004 |
|  | 8601309 |
|  | 8615004 |
|  | 8622692 |
|  | 8709193 |
|  | 8797825 |
|  | 8940099 |
|  | 9060679 |
|  | 9214631 |
|  | 9244310 |
|  | 9252186 |
|  | 9334723 |
|  | 9372968 |
|  | 9409737 |
|  | 9452483 |
|  | 9566883 |
|  | 9721103 |
|  | 9734360 |
|  | 9738011 |
|  | 9792645 |
|  | 9859996 |
|  | 9865694 |
|  | 9873017 |
|  | 9882613 |
|  | 9892650 |
|  | 9990853 |
|  | 12459277 |
|  | 12711254 |
|  | 15310755 |
|  | 15169890 |
|  | 16051172 |
|  | 16109975 |
|  | 10882136 |
|  | 15489334 |
|  | 16540234 |
|  | 17284228 |
|  | 17307728 |
|  | 17316064 |
|  | 17318773 |
|  | 17351338 |
|  | 17354114 |
|  | 17463416 |
|  | 17701919 |
|  | 8657102 |
|  | 9865693 |
|  | 12872135 |
|  | 17098744 |
|  | 17202411 |
|  | 17237376 |
|  | 17273479 |
|  | 17310217 |
|  | 17392377 |
|  | 17486138 |
|  | 17692539 |
|  | 1588976 |
|  | 15946255 |
|  | 16875840 |
|  | 16928772 |

---

|  |  |
| --- | --- |
| CellType | cells in white pulp |
|  | glandular cells |
|  | Hepatocytes |
|  | squamous epithelial cells |
|  | respiratory epithelial cells |
|  | trophoblastic cells |
|  | Macrophages |
|  | cells in granular layer |
|  | Pneumocytes |
|  | cells in molecular layer |
|  | cells in seminiferus ducts |
|  | germinal center cells |
|  | myocytes |
|  | Purkinje Cells |
|  | Leydig Cells |
|  | non-germinal center cells |
|  | glial cells |
|  | neuronal cells |
|  | cells in glomeruli |
|  | follicle cells |
|  | cells in tubules |
|  | lymphoid tissue |
|  | urothelial cells |
|  | hematopoietic cells |
|  | cells in endometrial stroma |
|  | epidermal cells |
|  | ovarian stroma cells |
|  | smooth muscle cells |
|  | exocrine glandular cells |
|  | cells in red pulp |
|  | islets of Langerhans |
|  | bile duct cells |
|  | hepatocyte |
|  | squamous cell |
|  | airway epithelial cell |
|  | trophoblast |
|  | macrophage |
|  | pneumocyte |
|  | germinal center B-cell |
|  | muscle cell |
|  | Purkinje cell |
|  | Leydig cell |
|  | neuroglia |
|  | neuron |
|  | ovary follicle cell |
|  | kidney tubule cell |
|  | transitional epithelial cell |
|  | hematopoietic cell |
|  | endometrial stromal cell |
|  | epidermis cell |
|  | smooth muscle myocyte |
|  | exocrine cell |

---

|  |  |
| --- | --- |
| Tissue | spleen |
|  | parathyroid gland |
|  | Liver |
|  | esophagus |
|  | bronchus |
|  | stomach, lower |
|  | Placenta |
|  | Lung |
|  | fallopian tube |
|  | Cerebellum |
|  | stomach, upper |
|  | Prostate |
|  | gall bladder |
|  | testis |
|  | rectum |
|  | lymph node |
|  | heart muscle |
|  | Salivary gland |
|  | hippocampus |
|  | cerebral cortex |
|  | thyroid gland |
|  | seminal vesicle |
|  | nasopharynx |
|  | tonsil |
|  | skeletal muscle |
|  | oral mucosa |
|  | Kidney |
|  | cervix, uterine |
|  | Adrenal gland |
|  | Ovary |
|  | Appendix |
|  | lateral ventricle |
|  | colon |
|  | urinary bladder |
|  | duodenum |
|  | bone marrow |
|  | uterus, post-menopause |
|  | breast |
|  | uterus, pre-menopause |
|  | Skin |
|  | vagina |
|  | small intestine |
|  | vulva/anal skin |
|  | smooth muscle |
|  | Pancreas |
|  | epididymis |
|  | myocardium |
|  | skeletal muscles |
|  | mouth mucosa |
|  | lymphoid tissue |

---

|  |  |
| --- | --- |
| Organism | Homo sapiens {Organism urn:agi-taxid:9606} |
|  | Mus musculus {Organism urn:agi-taxid:10090} |
|  | Rattus norvegicus {Organism urn:agi-taxid:10116} |
|  | Homo sapiens |
|  | Mus musculus |
|  | Rattus norvegicus |
|  | Bos taurus |
|  | Danio rerio |
|  | Human |

---

|  |  |
| --- | --- |
| Mouse chromosome position | 12 |
|  | 12 C1-C3 |

---

|  |  |
| --- | --- |
| OMIM ID | 164008 |
|  | 612132 |
|  | OMIM:164008 |

---

|  |  |
| --- | --- |
| Rat chromosome position | 6q23 |

---

|  |  |
| --- | --- |
| Hugo ID | 7797 |
|  | HGNC:7797 |

---

|  |  |
| --- | --- |
| Human chromosome position | 14q13.2 |
|  | 14q13 |

---

|  |  |
| --- | --- |
| Swiss-Prot Accession | P25963 |
|  | P25963.1 |
|  | Q9Z1E3 |
|  | Q9Z1E3.2 |
|  | Q63746 |
|  | Q63746.1 |
|  | B2R8L6 |
|  | Q3U9W9 |
|  | Q3UB40 |
|  | Q80ZX5 |
|  | B2RYS5 |
|  | Q9UGJ5 |
|  | IKBA\_HUMAN |
|  | IKBA\_MOUSE |
|  | IKBA\_RAT |
|  | B2R8L6\_HUMAN |
|  | B2RYS5\_RAT |
|  | Q3UB40\_MOUSE |

---

|  |  |
| --- | --- |
| PIR ID | A39935 |
|  | A44437 |

---

|  |  |
| --- | --- |
| GenBank ID | NC\_000014 |
|  | NM\_020529 |
|  | NP\_065390 |
|  | NG\_007571 |
|  | AJ249283 |
|  | AJ249284 |
|  | AJ249285 |
|  | AJ249286 |
|  | AJ249287 |
|  | CAB65110 |
|  | AJ249288 |
|  | CAB65111 |
|  | AJ249289 |
|  | CAB65112 |
|  | AJ249290 |
|  | CAB65113 |
|  | AJ249291 |
|  | CAB65114 |
|  | AJ249294 |
|  | CAB65556 |
|  | AJ249295 |
|  | AL133163 |
|  | AY496422 |
|  | AAR29599 |
|  | CH471078 |
|  | EAW65875 |
|  | CS542431 |
|  | CAM91165 |
|  | HI574170 |
|  | CBX47574 |
|  | KT584919 |
|  | S67380 |
|  | AAD14949 |
|  | U08468 |
|  | AK311515 |
|  | AK313421 |
|  | BAG36213 |
|  | AY033600 |
|  | AAK51149 |
|  | BC002601 |
|  | AAH02601 |
|  | BC004983 |
|  | AAH04983 |
|  | BT007091 |
|  | AAP35754 |
|  | BU737609 |
|  | M69043 |
|  | AAA16489 |
|  | P25963 |
|  | NC\_000078 |
|  | NM\_010907 |
|  | NP\_035037 |
|  | AC158398 |
|  | CH466526 |
|  | EDL36726 |
|  | U57524 |
|  | AAD10341 |
|  | AI462015 |
|  | AK089215 |
|  | AK151115 |
|  | BAE30124 |
|  | AK151608 |
|  | BAE30547 |
|  | AK161622 |
|  | BC046754 |
|  | AAH46754 |
|  | CF379853 |
|  | U36277 |
|  | AAA79696 |
|  | Q9Z1E3 |
|  | NC\_005105 |
|  | NM\_001105720 |
|  | NP\_001099190 |
|  | AC\_000074 |
|  | AABR07064338 |
|  | AAHX01044232 |
|  | AAHX01044233 |
|  | CH473947 |
|  | EDM03427 |
|  | EDM03428 |
|  | BC166886 |
|  | AAI66886 |
|  | BF417507 |
|  | CA509173 |
|  | CB316505 |
|  | CB324027 |
|  | CB793714 |
|  | FQ214065 |
|  | FQ215246 |
|  | FQ225350 |
|  | FQ225877 |
|  | FQ226288 |
|  | FQ227283 |
|  | FQ233200 |
|  | FQ233557 |
|  | FQ233627 |
|  | FQ234138 |
|  | FQ234262 |
|  | X63594 |
|  | CAA45138 |
|  | Q63746 |
|  | ABBA01061503 |
|  | AAHY01100242 |
|  | AAHY01100243 |
|  | AC\_000146 |
|  | AC\_000034 |
|  | NC\_018925 |
|  | AMYH02029008 |
|  | AABR06044120 |
|  | AABR06044119 |
|  | NT\_026437 |
|  | NW\_001838110 |
|  | NW\_925539 |
|  | AC\_000057 |
|  | CR589981 |
|  | CR591996 |
|  | CR592525 |
|  | CR593002 |
|  | CR594547 |
|  | CR595601 |
|  | CR596344 |
|  | CR600730 |
|  | CR603242 |
|  | CR604111 |
|  | CR606719 |
|  | CR610344 |
|  | CR610623 |
|  | CR612243 |
|  | CR613814 |
|  | CR615977 |
|  | CR617669 |
|  | CR618438 |
|  | CR621120 |
|  | CR623392 |
|  | CR626144 |
|  | Q9UGJ5 |
|  | NT\_039551 |
|  | NW\_001030500 |
|  | Q3U9W9 |
|  | Q3UB40 |
|  | NW\_001084845 |
|  | NW\_047760 |
|  | XM\_343065 |
|  | XP\_343066 |
|  | XM\_001075778 |
|  | XP\_001075778 |
|  | BG078274 |
|  | NW\_000053 |
|  | BG064981 |
|  | 14548069 |
|  | 57674 |
|  | 1022734 |
|  | 123787735 |
|  | 123791056 |
|  | 28386026 |
|  | 4097335 |
|  | 61226507 |
|  | 74185295 |
|  | 74195979 |
|  | 119586279 |
|  | 126682 |
|  | 12803543 |
|  | 13436416 |
|  | 14029101 |
|  | 187291 |
|  | 30583021 |
|  | 39654021 |
|  | 6688192 |
|  | 6706211 |
|  | 74753317 |
|  | NT\_086806 |
|  | 17221416 |
|  | 75066005 |
|  | 86823904 |
|  | AAI05485 |
|  | AJ420928 |
|  | BC105484 |
|  | CAD12786 |
|  | NC\_007319 |
|  | NM\_001045868 |
|  | NP\_001039333 |
|  | NW\_001494046 |
|  | Q8WNW7 |
|  | 29476855 |
|  | 37725734 |
|  | 38382969 |
|  | 82201956 |
|  | 82209677 |
|  | AAH50175 |
|  | AAH62524 |
|  | AAO26406 |
|  | AI545462 |
|  | AI584648 |
|  | AY163841 |
|  | BC050175 |
|  | BC062524 |
|  | BX649530 |
|  | NC\_007128 |
|  | NM\_199629 |
|  | NP\_955923 |
|  | NW\_001511519 |
|  | Q6K195 |
|  | Q7ZW68 |
|  | 148704779 |
|  | 149051254 |
|  | 149051255 |
|  | XM\_234230 |
|  | XP\_234230 |
|  | NW\_043949 |
|  | NT\_037845.1 |
|  | 54261748 |
|  | 126007125 |
|  | 49659397 |
|  | 54261749 |
|  | 323099 |
|  | BC050175,BC062524 |
|  | Dr.77409 |
|  | ZDB-GENE-030131-1819 |
|  | dre:323099 |

---

|  |  |
| --- | --- |
| Swiss-Prot ID | IKBA\_HUMAN |
|  | IKBA\_MOUSE |
|  | IKBA\_RAT |
|  | Q3UB40\_MOUSE |
|  | B2RYS5\_RAT |
|  | B2R8L6\_HUMAN |

---

|  |  |
| --- | --- |
| Cell Localization | Cytoplasm |
|  | Nucleus |

---

|  |  |
| --- | --- |
| Ensembl ID | ENSG00000100906 |
|  | ENSP00000216797.5 |
|  | ENST00000216797.9 |
|  | ENSMUSG00000021025 |
|  | ENSMUSP00000021413.7 |
|  | ENSMUST00000021413.8 |
|  | ENSRNOG00000007390 |
|  | ENSRNOP00000009894.5 |
|  | ENSRNOT00000009894.6 |
|  | ENSMUST00000021413 |
|  | ENSRNOT00000009894 |
|  | ENSRNOP00000009894 |
|  | ENSMUSP00000021413 |
|  | ENSP00000216797 |
|  | ENST00000216797 |

---

|  |  |
| --- | --- |
| MGI ID | MGI:104741 |
|  | 104741 |
|  | 2144835 |

---

|  |  |
| --- | --- |
| RGD ID | 3171 |

---

|  |  |
| --- | --- |
| Unigene ID | Hs.81328 |
|  | Mm.170515 |
|  | Rn.12550 |
|  | Bt.9027 |
|  | Dr.77409 |
|  | Rn.104476 |

---

|  |  |
| --- | --- |
| Homologene ID | 7863 |

---

|  |  |
| --- | --- |
| Shape | O-vertex |

---

|  |  |
| --- | --- |
| IPI ID | IPI00211841 |
|  | IPI00331017 |
|  | IPI00018330 |

---

|  |  |
| --- | --- |
| Proteome Biochemical Function | Inhibitor or repressor |

---

|  |  |
| --- | --- |
| Microarray ID | 101554\_at |
|  | 104149\_at |
|  | X63594cds\_at |
|  | X63594cds\_g\_at |
|  | A\_42\_P544487 |
|  | 206329\_Rn |
|  | Msa.1690.0\_at |
|  | Msa.1690.0\_g\_at |
|  | 1448306\_at |
|  | 133630\_r\_at |
|  | 1438157\_s\_at |
|  | 167965\_f\_at |
|  | A\_65\_P03979 |
|  | H3026A08-3 |
|  | A\_51\_P295192 |
|  | NM\_010907 |
|  | 201502\_s\_at |
|  | 231699\_at |
|  | 1461\_at |
|  | M69043\_at |
|  | A\_23\_P106002 |
|  | I\_959455 |
|  | A\_14\_P120088 |
|  | A\_14\_P114684 |
|  | A\_14\_P133399 |
|  | 1420088\_at |
|  | 1449731\_s\_at |

---

|  |  |
| --- | --- |
| Homo sapiens Chromosome position | 14q13 |

---

|  |  |
| --- | --- |
| Ensembl Transcript ID | ENST00000216797 |

---

|  |  |
| --- | --- |
| FunctionalClass | NF-kappaB binding |
|  | heat shock protein binding |
|  | nuclear localization sequence binding |
|  | protein binding |
|  | ubiquitin protein ligase binding |
|  | transcription factor binding |

---

|  |  |
| --- | --- |
| Bos taurus Chromosome position | 21 |

---

|  |  |
| --- | --- |
| Mus musculus Chromosome position | 12 C1-C3 |
|  | 12 |

---

|  |  |
| --- | --- |
| Proteome Subcellular Localization | Cytoplasmic |

---

|  |  |
| --- | --- |
| Compartment | cytoplasm |
|  | DNA |
|  | void |

---

|  |  |
| --- | --- |
| KEGG pathway | Adipocytokine signaling pathway |
|  | Apoptosis |
|  | B cell receptor signaling pathway |
|  | Chronic myeloid leukemia |
|  | Small cell lung cancer |
|  | T cell receptor signaling pathway |
|  | Toll-like receptor signaling pathway |

---

|  |  |
| --- | --- |
| RefSeq ACCNUM | NP\_065390 |

---

|  |  |
| --- | --- |
| Rattus norvegicus Chromosome position | 6q23 |

---

|  |  |
| --- | --- |
| Ensembl Gene ID | ENSG00000100906 |
|  | LRG\_89 |

---

|  |  |
| --- | --- |
| Keyword | cytoplasm |
|  | ubiquitin |
|  | nucleus |
|  | DNA |
|  | binding activity |
|  | kinetochore |

---

|  |  |
| --- | --- |
| LocatorString | pmquery:curcumin+prostate+cancer |
|  | pmquery:curcumin prostate cancer |

---

|  |  |
| --- | --- |
| Proteome Organismal Role | Cell death/Apoptosis |
|  | Anti-pathogen response |

---
